# Intracellular lipid surveillance: Modulating protein dynamics through lipid sensing

**DOI:** 10.1002/ctm2.1147

**Published:** 2022-12-19

**Authors:** Abigail Watterson, Peter M. Douglas

**Affiliations:** ^1^ Department of Molecular Biology UT Southwestern Medical Center Dallas Texas USA; ^2^ Hamon Center for Regenerative Science and Medicine UT Southwestern Medical Center Dallas Texas USA

1

Lipids play fundamental roles in nearly all biological processes. While this class of macromolecules is expansive and complex, lipid roles in the cell can be confined to three major functions: membrane composition, signalling and energetic reserve. In cellular and organellular membranes, lipids provide structural integrity, compositional specificity and compartmentalisation. In the maintenance of cellular and metabolic homeostasis, lipid‐derived hormones serve as regulatory ligands in signal transduction pathways that promote growth and development.^1^ The sterol lipid, cholesterol, serves as both an important membrane component and the precursor for most steroid hormones.^2^ Noteworthy discoveries involving the regulation of lipids include the identification and characterisation of cholesterol sensing through the sterol regulatory element binding protein (SREBP), which couples sterol availability with membrane composition and steroidogenesis.^3^ When cholesterol availability is limited, SREBP promotes lipogenesis to increase sterol production and restore membrane fluidity. This seminal discovery initiated our understanding of sterol lipid sensing and represented the first molecular mechanism for regulating lipid availability. However, how cells measure their lipid availability in the context of maintaining energetic reserves remains an important yet outstanding question. Specifically, it was unclear how a starving cell detects and responds to the depletion of its intracellular lipid pools.

Similar to lipid storage in adipose tissue, nearly every cell in the body stores lipids in the form of lipid droplets. These lipid reservoirs provide cells an intrinsic source of carbon‐rich molecules that can be rapidly utilised as substrates for mitochondrial respiration and downstream energy production, in the form of adenosine triphosphate (ATP), upon metabolic demand.^4^ Energy released from ATP hydrolysis is essential for nearly every biological process and therefore requires tight regulation to ensure cellular survival. Since cells continuously metabolise their lipid stores for energy production, they must simultaneously replenish metabolic resources through nutrient absorption. When extracellular resources become limited, as with reduced systemic nutrient availability or in tissues with limited blood flow, cells must be able to detect lipid depletion and initiate a response to both increase nutrient intake and restore metabolic homeostasis. While such mechanisms had not previously been described, a recent study published in *Nature* defined a novel intracellular lipid surveillance pathway that allows intestinal cells in the roundworm, *Caenorhabditis elegans*, to sense and respond to lipid depletion.^5^

Watterson et al. implicated a single de novo‐synthesised lipid, geranylgeranyl pyrophosphate (GGPP), as a cellular indicator of total lipid availability through its ability to sequester and inactivate the starvation‐activated transcriptional regulator, nuclear hormone receptor 49 (NHR‐49),^6^ in the cytosol.^5^ This study demonstrates that under conditions of ample resources, NHR‐49, the *C. elegans* orthologue of mammalian nuclear receptors, hepatic nuclear factor 4 and peroxisome proliferator‐activated receptor, is retained in the cytosol in an inactive state through its physical association with endocytic transport vesicles. To remain associated with the vesicle, NHR‐49 requires the prenol lipid, GGPP, and its conjugation to the small G protein, RAB‐11.1. Synthesis of GGPP via the mevalonate/isoprenoid pathway requires intracellular carbon resources such as those heavily represented in lipid droplets, which are ultimately metabolised to acetyl‐coenzyme A , the two‐carbon precursor for this biosynthetic pathway^7^ (Figure 1). When intracellular lipids become limited, cells lack the appropriate carbon‐rich resources needed to synthesise GGPP, thereby preventing NHR‐49 vesicular retention and allowing for its translocation into the nucleus. Once nuclear, NHR‐49 activates a transcriptional program to restore metabolic homeostasis in the cell.

**FIGURE 1:**
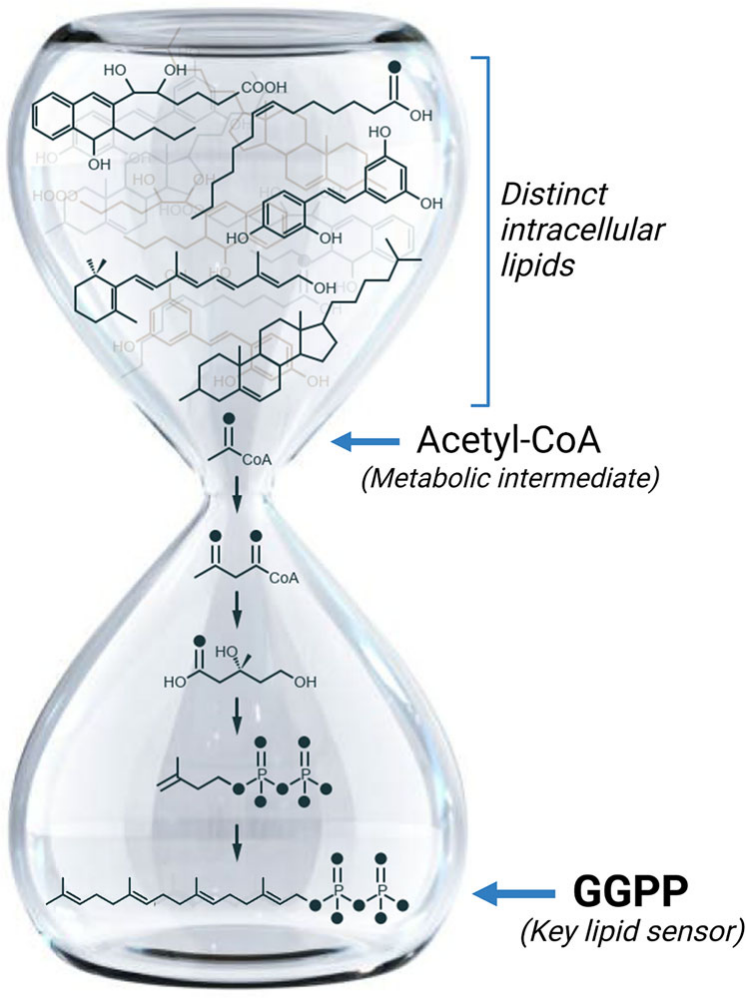
Model for intracellular lipid surveillance by measuring the synthesis of a single lipid, geranylgeranyl pyrophosphate (GGPP), from the metabolic intermediate, acetyl‐coenzyme A (CoA), as an indicator of overall lipid availability

In addition to promoting fatty acid breakdown via β‐oxidation, NHR‐49 increases nutrient intake by activating expression of genes encoding cell‐surface nutrient transporters and proteins that facilitate membrane trafficking. In this fashion, the cell not only produces more nutrient transporters but also ensures their proper trafficking to and retention on the cell surface. Coupled with the upregulation of genes involved in the breakdown of these resources, the data presented in this study suggest that activation of this stress‐responsive intracellular lipid surveillance pathway provides a means for starving epithelial cells to restore their essential lipid levels. In summary, GGPP sensing by NHR‐49 enables cells to monitor their metabolisable lipid pool and initiate a response to restore metabolic homeostasis upon lipid depletion. Moreover, this study demonstrates how lipid availability can impact membrane protein dynamics by mediating the trafficking and residency of proteins on the cell surface (Figure 2).

**FIGURE 2:**
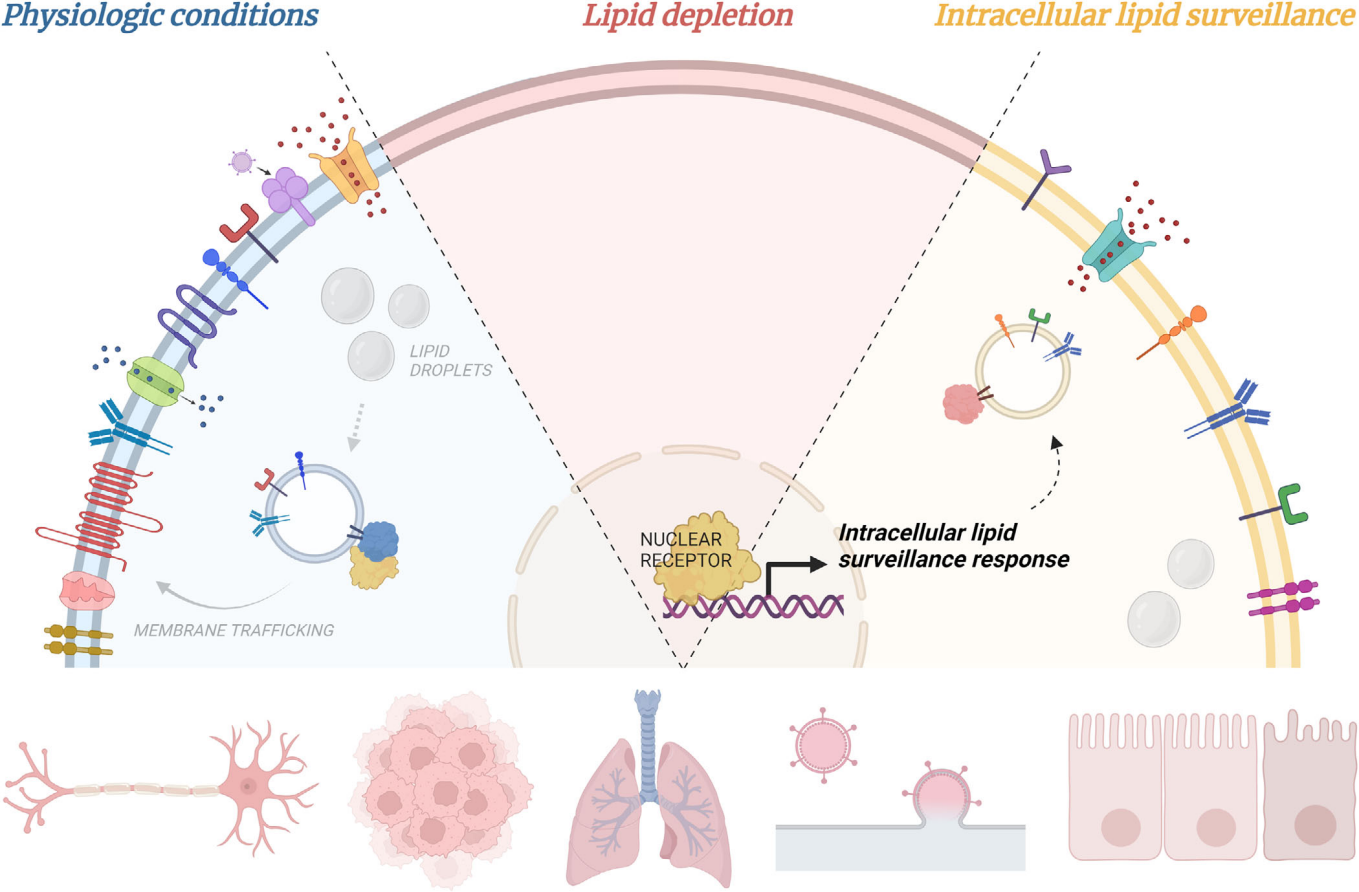
Modulating cell surface protein residency through intracellular lipid surveillance enables cellular adaption to metabolic stress caused by lipid depletion and offers biomedical potential. Schematic demonstrates lipid‐dependent trafficking and residency of membrane proteins. Upon lipid depletion, nuclear hormone receptor 49 (NHR‐49) enters the nucleus and activates the intracellular lipid surveillance response to restore membrane protein recycling and metabolic homeostasis. Created with biorender.com

Although reported in the worm intestinal epithelium, there is evidence that intracellular lipid surveillance may also occur in humans. In particular, studies investigating the molecular effects of statin treatment in human cells have hinted at potential pathway conservation. Despite the cholesterol‐lowering effects of statin treatment via inhibition of cholesterol biosynthesis, the enzymatic target of statins is also required for isoprenoid synthesis. As such, reduced GGPP synthesis by statins impairs Rab GTPase‐mediated trafficking of membrane proteins by preventing their ability to bind to transport vesicles. In turn, statin treatment has been shown to significantly reduce the membrane residency of essential cell surface proteins, such as ion channels in cardiomyocytes or dopamine transporters in the brains of patients with Parkinson's disease.^8^, ^9^ These studies indicate that statin‐mediated inhibition of isoprenoid synthesis may be detrimental to the cell. However, *Rab11a*, the mammalian homolog of the gene involved in membrane protein recycling that is activated by intracellular lipid surveillance in the worm, is significantly upregulated upon statin treatment in primary human microvascular endothelial cells,^5^, ^10^ suggesting that these cells are attempting to circumvent the trafficking defects caused by statins by increasing membrane protein recycling. Thus, the cellular stress response described in Watterson et al. may similarly occur in human cells as a means to ensure proper localisation of membrane proteins involved in important cellular functions and events.

While the findings presented in Watterson et al. are just the beginning of what researchers are hopeful will become an impactful and medically relevant field, one can speculate how further understanding of this lipid‐sensing mechanism could yield future therapeutic interventions. In addition to this study's implications in understanding how individual cells control their fat storage, the intracellular lipid surveillance pathway offers potential as a therapeutic target for disorders and ailments resulting from aberrant cell‐surface residency of important receptors and regulatory proteins. Examples of such membrane proteins include regulators of synapse function and maintenance in neurodegenerative diseases, growth factors or antigens on cancer cells, molecular machinery required for viral entry and exit, the chloride‐ion channel, CFTR, on the surface of lung epithelia in cystic fibrosis, as well as nutrient transporters and cell junction proteins in the context of enteric dysfunction (Figure 2). As the lipid‐sensing pathway described in Watterson et al. represents an inducible mechanism for cells to increase steady‐state expression of membrane proteins, targeting this adaptive response in human cells could provide a novel means of controlling membrane residency of disease‐linked proteins. Thus, in addition to its function in promoting nutrient absorption when lipids become depleted, the intracellular lipid surveillance pathway harbours tremendous biomedical potential.

## CONFLICT OF INTEREST

The authors declare they have no conflicts of interest.

## References

[1] Chawla A , Repa JJ , Evans RM , Mangelsdorf DJ . Nuclear receptors and lipid physiology: opening the X‐files. Science. 2001;294:1866‐1870. doi:10.1126/science.294.5548.1866 11729302

[2] Rone MB , Fan J , Papadopoulos V . Cholesterol transport in steroid biosynthesis: role of protein–protein interactions and implications in disease states. Biochim Biophys Acta. 2009;1791:646‐658. doi:10.1016/j.bbalip.2009.03.001 19286473 PMC2757135

[3] Brown MS , Goldstein JL . The SREBP pathway: regulation of cholesterol metabolism by proteolysis of a membrane‐bound transcription factor. Cell. 1997;89:331‐340. doi:10.1016/s0092-8674(00)80213-5 9150132

[4] Walther TC . Lipid droplets and cellular lipid metabolism. Annu Rev Biochem. 2012;81:687‐714. doi:10.1146/annurev-biochem-061009-102430 22524315 PMC3767414

[5] Watterson A , Tatge L , Wajahat N , et al. Intracellular lipid surveillance by small G protein geranylgeranylation. Nature. 2022;605:736‐740. doi:10.1038/s41586-022-04729-7 35585236 PMC9885440

[6] Van Gilst MR , Hadjivassiliou H , Jolly A , Yamamoto KR . Nuclear hormone receptor NHR‐49 controls fat consumption and fatty acid composition in *C. elegans* . PLoS Biol. 2005;3:e53. doi:10.1371/journal.pbio.0030053 15719061 PMC547972

[7] Goldstein JL , Brown MS . Regulation of the mevalonate pathway. Nature. 1990;343:425‐430. doi:10.1038/343425a0 1967820

[8] Ronzier E , Parks XX , Qudsi H , Lopes CM . Statin‐specific inhibition of Rab‐GTPase regulates cPKC‐mediated IKs internalization. Sci Rep. 2019;9:17747. doi:10.1038/s41598-019-53700-6 31780674 PMC6882895

[9] Jeong SH , Lee HS , Chung SJ , et al. Effects of statins on dopamine loss and prognosis in Parkinson's disease. Brain. 2021;144:3191‐3200. doi:10.1093/brain/awab292 34347020

[10] Boerma M , Fu Q , Wang J , et al. Comparative gene expression profiling in three primary human cell lines after treatment with a novel inhibitor of Rho kinase or atorvastatin. Blood Coagul Fibrinol. 2008;19:709‐718. doi:10.1097/MBC.0b013e32830b2891 PMC2713681 18832915

